# Fiji plugins for qualitative image annotations: routine analysis and application to image classification

**DOI:** 10.12688/f1000research.26872.2

**Published:** 2021-02-12

**Authors:** Laurent S. V. Thomas, Franz Schaefer, Jochen Gehrig

**Affiliations:** 1 Acquifer Imaging GmbH, Heidelberg, Germany; 2 DITABIS, Digital Biomedical Imaging Systems AG, Pforzheim, Germany; 3 Department of Pediatrics, University Children’s Hospital, Heidelberg, Germany

**Keywords:** ImageJ, Fiji, KNIME, image annotation, image classification, ground-truth labelling, qualitative analysis, bioimage analysis

## Abstract

Quantitative measurements and qualitative description of scientific images are both important to describe the complexity of digital image data. While various software solutions for quantitative measurements in images exist, there is a lack of simple tools for the qualitative description of images in common user-oriented image analysis software. To address this issue, we developed a set of Fiji plugins that facilitate the systematic manual annotation of images or image-regions. From a list of user-defined keywords, these plugins generate an easy-to-use graphical interface with buttons or checkboxes for the assignment of single or multiple pre-defined categories to full images or individual regions of interest. In addition to qualitative annotations, any quantitative measurement from the standard Fiji options can also be automatically reported. Besides the interactive user interface, keyboard shortcuts are available to speed-up the annotation process for larger datasets. The annotations are reported in a Fiji result table that can be exported as a pre-formatted csv file, for further analysis with common spreadsheet software or custom automated pipelines. To illustrate possible use case of the annotations, and facilitate the analysis of the generated annotations, we provide examples of such pipelines, including data-visualization solutions in Fiji and KNIME, as well as a complete workflow for training and application of a deep learning model for image classification in KNIME. Ultimately, the plugins enable standardized routine sample evaluation, classification, or ground-truth category annotation of any digital image data compatible with Fiji.

## Introduction

A common requirement of most imaging projects is to qualitatively describe images, either by assigning them to defined categories or by selecting a set of descriptive keywords. This routine task is shared by various scientific fields, for instance in biomedical research for the categorization of samples, in clinical imaging for image-based diagnostics, or in manufacturing for the description of object-properties.

Qualitative descriptors, or keywords, can correspond to the presence or discrete count of features, the evaluation of quality criteria, or the assignment of images to specific categories. While automated methods for such qualitative description may exist, they usually require substantial effort for their implementation and validation. Therefore, for routine image data analysis and inspection, the qualitative description is usually performed manually. Similarly, for the training of machine learning models, manual annotations by experts are typically used as ground truth material.

For small datasets of a few dozen images, manual description of images can be performed by reporting the image identifier and qualitative descriptors in a simple spreadsheet. However, for larger datasets or large number of descriptors, this becomes quickly overwhelming and error prone as one needs to inspect a multitude of images while appending information to increasingly complex tables. Several software tools have been reported for the annotation of images or regions of interest (ROIs), mostly targeting ground-truth annotations for automated classification and object-detection (see Hollandi *et al.*, 2020 for a comparison of available solutions). However, most of these software tools have been initially designed for the annotation of real-life photographs, and thus have limited compatibility with scientific image formats (e.g. 16-bit tiff), besides requiring specific installation and configuration. Most bioimage analysis software packages similarly support annotations in the form of regions of interests (ROIs) associated to a category label (e.g. ImageJ/Fiji ( Schindelin *et al.*, 2012; Schneider *et al.*, 2012), QuPath ( Bankhead *et al.*, 2017), Ilastik ( Berg *et al.*, 2019), ICY ( de Chaumont *et al.*, 2012), KNIME ( Berthold *et al.*, 2009)), with applications for classification or segmentation. Typically, with those existing solutions, only a single category descriptor can be associated to an image or ROI. There is surprisingly no widespread solution available in common user-oriented scientific image analysis software, for the assignment of multiple descriptive keywords to images or ROIs. We previously proposed a standalone python annotation tool for this purpose, illustrated with the annotation of zebrafish morphological phenotypes ( Westhoff *et al.*, 2020). However, we believe that a similar implementation integrated within a widespread scientific image-analysis software would improve compatibility with image formats, software distribution, long-term support and adoption by the community. Therefore, we developed a set of plugins for Fiji, to facilitate and standardize routine qualitative image annotations, in particularly for large image datasets. We also illustrate possible applications of the resulting standardized qualitative description for the visualization of data-distribution, or the training of supervised image classification models.

## Methods

### Implementation

**Figure 1.**
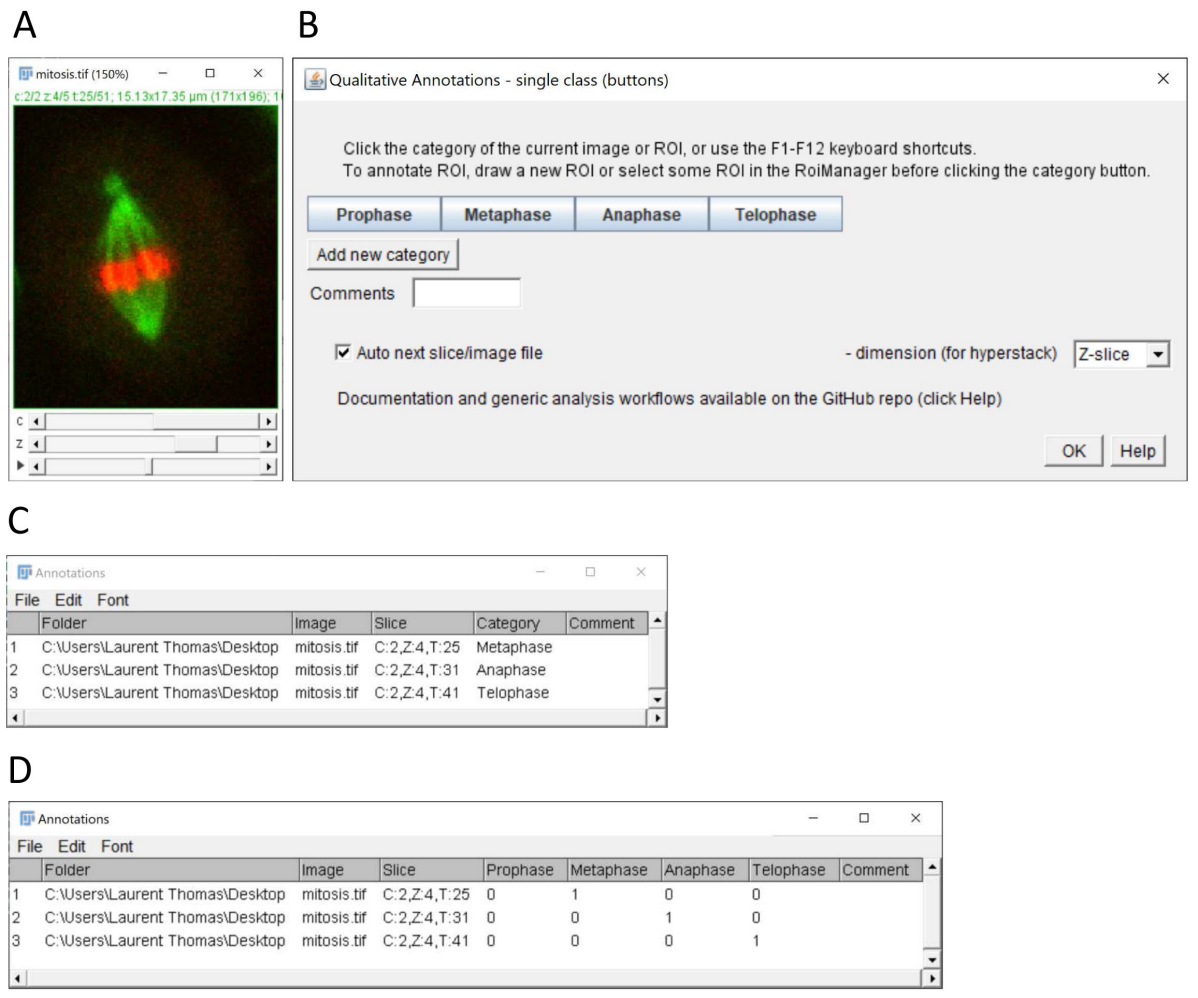
Single-category annotation of images in multi-dimensional stacks. ( **A**) Example of multi-dimensional image stack used to annotate mitotic stages in time-lapse data (source: ImageJ example image “Mitosis” – image credit NIH). ( **B**) Graphical interface of the *single class (buttons)* plugin configured for annotation of 4 mitotic stages. ( **C**) Results table with annotated categories (column *category*) generated by the plugin after selecting the *single category column* option in the plugin configuration window (not shown). ( **D**) Alternative results table output using 1-hot encoding after selecting the option *1 column per category*. The resulting 1-hot encoding of categories can be used for the training of classification algorithms.

We developed a set of Fiji plugins for the assignment of single or multiple descriptive keywords to images, or image-regions outlined by ROIs. The plugins provide an intuitive graphical user-interface (GUI) consisting of either buttons, checkboxes, or dropdown menus for the assignment of user-defined keywords ( Figure 1– Figure 3). The GUI is automatically generated from a set of keywords, defined at the beginning of the annotation session. Additional keywords and associated GUI elements can be later added to the plugin interface during the annotation to account for new descriptors, by clicking the “Add category” button. In addition to the pre-defined set of keywords, arbitrary image-specific comments can also be entered via a text input field. Furthermore, if *run Measure* is selected in the graphical interface, quantitative measurements as defined in Fiji’s menu *Analyze>Set Measurements* are reported in addition to the selected keywords. By default, the annotations and measurements are assigned to the entire image but can also describe image-regions outlined by ROI. The latter is simply achieved by drawing a new ROI on the image or selecting existing ROIs in the ROI Manager before assigning the keywords ( Figure 3). Newly drawn ROIs are automatically added to the ROI Manager upon annotation with the plugins.

**Figure 2.**
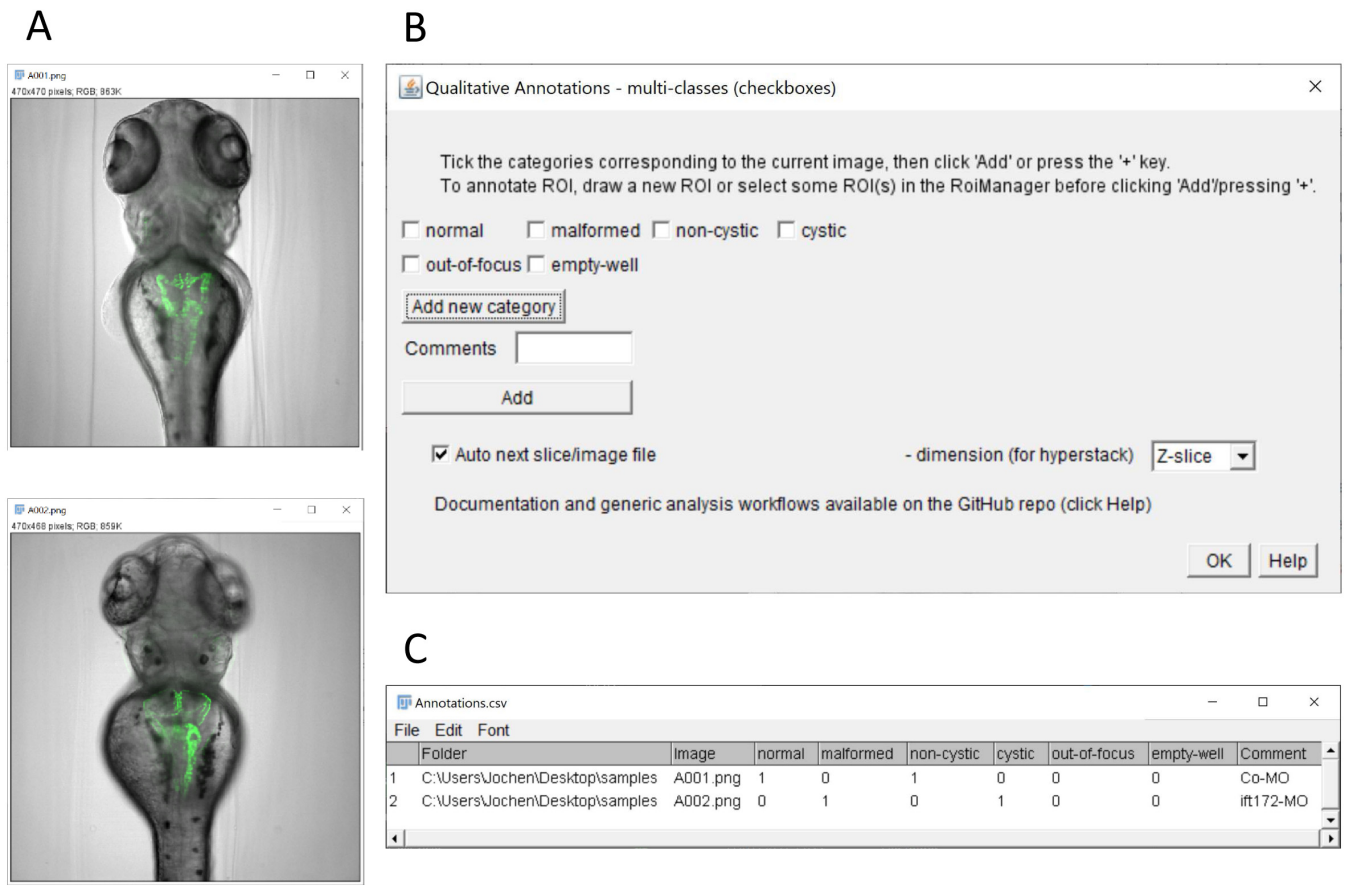
Annotation of multiple categories using the *multi-class (checkboxes)* plugin. ( **A**) Example images of transgenic zebrafish larvae of the *Tg(wt1b:egfp)* transgenic line after injection with control morpholino (upper panel) or with ift172 morpholino (lower panel) inducing pronephric cysts. In this illustration, the plugin is used to score overall morphology and cyst formation. It could also be used to mark erroneous images (such as out-of-focus or empty wells). Images are from ( Pandey *et al*., 2019). ( **B**) Graphical interface of the checkbox annotation plugin configured with 2 checkboxes for overall morphology, 2 checkboxes for presence of pronephric cysts, and checkboxes to report out-of-focus and empty wells. Contrary to the *single class (button)* plugin, multiple categories can be assigned to a given image. ( **C**) Resulting multi-category classification table with binary encoding of the annotations (True/False).

**Figure 3.**
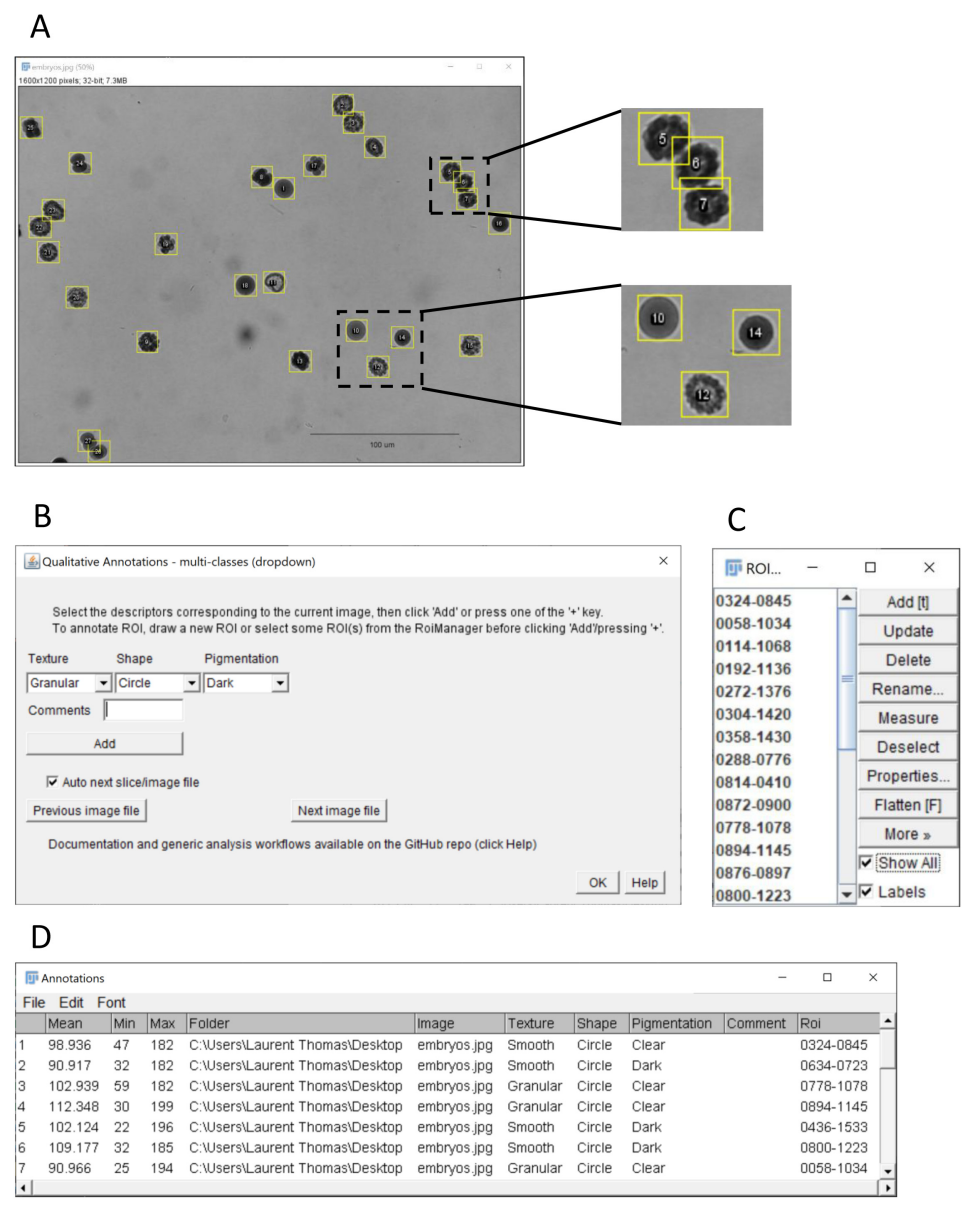
Qualitative and quantitative annotations of image regions using the *multi-class (dropdown)* plugin. ( **A**) ImageJ’s sample image “ *embryos*” after conversion to grayscale using the command *Image > Type > 32-bit* (image credit: NIH). The embryos outlined with yellow regions of interest were annotated using the “ *multi-class (dropdown)*” plugin. The insets at the top shows the annotation of overlapping ROIs, here corresponding to embryos with phenotype granular texture, dark pigmentation and elliptic shape. The inset at the bottom shows other embryos with different phenotypes (10: smooth/clear/circular, 12: granular/clear/elliptic, 14: smooth/dark/circular). ( **B**) Graphical interface of the multi-class (dropdown) plugin. Three exemplary features are scored for each embryo: texture (granular, smooth), shape (circle, ellipse) and pigmentation (dark, clear). Quantitative measurements as selected in the *Analyze > set Measurements* menu (here Mean, Min and Max grey level) are also reported for each embryo, when the *run Measure* option is ticked. ( **C**) ROI Manager with ROIs corresponding to annotated regions. ( **D**) Resulting classification table with the selected features, qualitative measurement and associated ROI identifier for the outlined embryos.

The selected keywords, comments, measurements, and the image filename and directory are reported in a Fiji result table window with one row per annotation event ( Figure 1C, D). An annotation event is triggered when a button is clicked, or a keyboard shortcut is pressed. With ROIs, the identifiers of the ROIs are also reported in the table, and the descriptors are saved as properties of the ROI objects. The information can then be retrieved from the ROIs using the Fiji scripting functions. The result table is updated row by row, as the user progresses with the annotations. Table rows can be deleted within Fiji if some annotations should be corrected. The result table can be saved as a csv file at any time and edited in a spreadsheet software or text editor, for instance to update the image directory column when images have been transferred to a different location or workstation.

Three plugins are provided to accommodate for different annotation modalities. The *single-class (buttons)* plugin ( Figure 1) allows the assignment of a single keyword per image by clicking the corresponding button. With this plugin, the user can decide if the result table should contain a single category column containing the clicked category keyword for each image ( Figure 1C), or one column per category with a binary code (0/1) depicting the assignment ( Figure 1D). The latter option is particularly suitable for the training of supervised classification algorithms, which typically expect for their training an array of probabilities with 1 for the actual image-category and 0 for all other categories (also called 1-hot encoding, see Müller & Guido, 2016).

The *multi-class (checkboxes)* plugin ( Figure 2) allows multiple keywords per image, which are selected via associated checkboxes ( Figure 2B). This yields a result table with one column per keyword, and a 0/1 code if the keywords apply or not ( Figure 2C). In this case, the table structure is similar to Figure 1D except that multiple keywords might be selected for a given image (i.e. multiple 1 for a given table row, as in row 1).

The *multi-class (dropdown)* plugin ( Figure 3) allows choosing keywords from distinct lists of choices using dropdown menus ( Figure 3B). The labels and choices for the dropdown menus are defined by the user in a simple csv file ( *Extended data*, Supplementary Figure 1) ( Thomas, 2020). This is convenient if multiple image features should be reported in separate columns (content, quality, etc.) with several options for each feature.

### Operation

The plugins run on any system capable of executing Fiji. Executing one of the plugins will first display a set of configuration windows to define the keywords, image browsing mode (stack or directory) and if quantitative measurements should be reported (“Run measure”). Upon validation of the configuration, the actual annotation interface as in Figure 1– Figure 3 is displayed and ready to use for annotation. For the *single-class (buttons)* plugin, annotations can be recorded by clicking the corresponding category button, or by pressing one of the F1-F12 keyboard shortcuts. The shortcuts are automatically mapped to the categories in the order of their respective buttons, e.g. pressing F1 is equivalent to clicking the leftmost button (see pop-up message when hovering the mouse over a button). For the *multi-class* plugins, the annotations can be recorded by clicking the *Add* button or pressing one of the *+* keys of the keyboard. For every plugin, an annotation event updates the results table as described in the implementation section, stores any newly drawn ROI into Fiji’s ROI Manager, and if the corresponding option is selected in the graphical user interface, the next image slice of the selected dimension is displayed in “stack” browsing mode when a stack is annotated. Similarly, in “directory” browsing mode the next image file is loaded. The annotations are automatically appended to an active Fiji table window if available. For Fiji installations with ImageJ versions below 1.53g, annotations will be appended to a table window entitled “Annotations” or “Annotations.csv” if available.

## Use cases

The described plugins allow the rapid and systematic description of single images, image-planes within multi-dimensional images or image-regions with custom keywords. Rich qualitative descriptions can thus be reported by combining multiple keywords, although the plugins can also be used for ground-truth category annotation, for which typically a single label is reported for each image instance. Additionally, by activating the measurement option, the qualitative description can be complemented by any of ImageJ’s quantitative measurements. The annotation tools can be used for routine image evaluation e.g. for the assignment of predefined categories, to identify outliers or low-quality images, or to assess the presence of a particular object or structure. Examples annotations are illustrated in Figure 1 (single-cell mitotic stage), Figure 2 (pronephric morphological alterations in transgenic zebrafish larvae ( Pandey *et al*., 2019) and Figure 3 (phenotypic description of multi-cellular embryos). Images and annotations are available as *Underlying data* ( Pandey *et al*., 2020).

For the annotation of ROIs, the presented plugins can be used in combination with our previously published *ROI 1-click tools* ( Thomas & Gehrig, 2020), which facilitate the creation of ROIs of predefined shapes, and the automated execution of custom commands for these ROIs. The generated ROIs can then be described with qualitative features using the hereby presented plugins, either for one ROI at a time, or by simultaneously selecting multiple ROIs. Besides facilitating qualitative annotations, the plugins have the advantage to generate tables with standardized structures that can potentially facilitate the visualization and analysis of the annotations by automated workflows.

**Figure 4.**
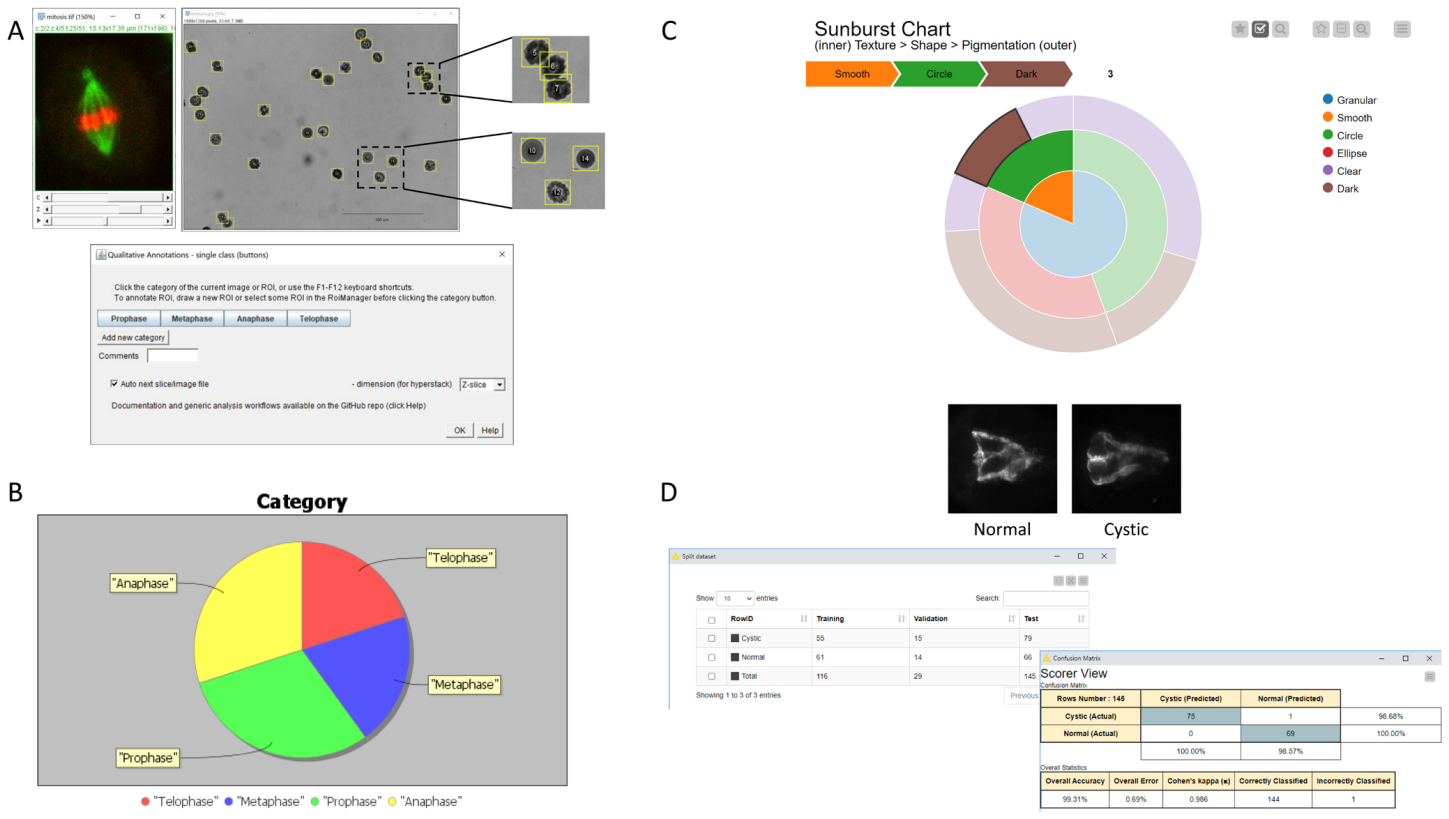
Overview of the annotation tools and possible use cases of the annotations. ( **A**) The qualitative annotation plugins provide simple graphical user interfaces for the annotations of images or image regions outlined by ROIs (see Figure 1– Figure 3). ( **B**) Pie chart visualization of the data-distribution from a single table column, here illustrated with the distribution of the mitotic stage in a population of cells (fictive distribution). The plot is generated in Fiji by the plugin “Pie chart from table column”, provided with the annotation plugins (see Supplementary Figure 2). ( **C**) Interactive sunburst chart visualization in KNIME, illustrated with the distribution of morphological phenotypes of multi-cellular embryos (as in Figure 3). Distinct data-columns are represented as successive levels of the chart (see Supplementary Figure 3). ( **D**) Training of a deep-learning model for image-classification in KNIME with representative images of the custom categories (kidney morphology in zebrafish larvae, left: normal, right: cystic), distribution of the annotated images between training, validation and test fraction, and result of the classification shown as a confusion matrix (see Supplementary Figure 4,5).

To illustrate and expand on the potential of such annotations, we provide a set of generic workflows which directly operate on tables generated by one of the presented plugins. The example workflows demonstrate classical scenarios for the exploitation of qualitative descriptors: (i) the visualization of data-distribution using a pie chart (Fiji plugin, Figure 4.B, Supplementary Figure 2), ii) interactive sunburst chart visualization ( *Extended data KNIME workflow*, Figure 4.C, Supplementary Figure 3) ( Thomas, 2020), and (iii) the training of a deep learning model for image classification (KNIME workflows, Figure 4.D, Supplementary Figure 4, 5). We developed a dedicated Fiji plugin for the pie chart visualization as part of the *Qualitative Annotations* update site. The plugin relies on the JFreeChart library, providing advanced customisation options and readily available for scripting in Fiji. The plugin allows representing the data-distribution from a table column and is macro-recordable. It is not limited to results tables generated with the annotation plugins, and thus offer a novel plotting option for end-users in Fiji. While the pie chart represents the distribution for a single data column, the sunburst chart visualization in KNIME allows visualizing and relating the distribution for multiple feature columns, as each column is represented as an additional level in the chart. Besides, the plot is generated by a javascript view node, which offers enhanced interactivity for the exploration of the data-distribution. Finally, for the training of a deep learning model for image-classification, we adapted an existing KNIME example workflow for transfer-learning of a pretrained model. We could demonstrate rapid model training and high classification accuracy with a moderate number of training images (116) for the classification of microscopy images representing kidney morphologies in developing zebrafish larvae ( Figure 4D, Supplementary Figure 4D). The KNIME workflows do not require advance knowledge of KNIME and can be readily used without major adaptation. By providing those examples, we also wish to facilitate and spread those advanced data-processing tools, by drastically reducing the need for custom development. We also provide detailed documentation about the workflows and required software dependencies on the GitHub repository ( https://github.com/LauLauThom/Fiji-QualiAnnotations).

## Conclusion

Here, we propose a set of plugins for the qualitative annotations of images or image regions, designed for the popular image analysis software Fiji. The annotations comprise user-defined keywords, as well as optional quantitative measurements as available in Fiji. The keywords can describe categorical classification, the evaluation of quality metrics or the presence of particular objects or structures. The plugins are easy to install and to use via an intuitive graphical user interface. In particular, the tools facilitate tedious qualitative annotation tasks, especially for large-datasets, or for the evaluation of multiple features. The annotations are recorded as standardized result tables, to facilitate automated analysis by generic workflows. To this extent, example workflows for data-visualization and supervised data classification are provided, which can be directly executed with the resulting annotation table without further customization effort ( Figure 4). Finally, video tutorials about the plugins and analysis workflows are available on YouTube.

## Data availability

### Underlying data

Zenodo: Fluorescently-labelled zebrafish pronephroi + ground truth classes (normal/cystic) + trained CNN model. https://doi.org/10.5281/zenodo.3997728 ( Pandey *et al*., 2020).

This project contains the following underlying data:

- Annotations-multiColumn.csv. (Ground-truth category annotations.)
- Annotations-singleColumn.csv. (Ground-truth category annotations.)
- images.zip. (Images of fluorescently labelled pronephroi in transgenic *Tg(wt1b:EGFP)* zebrafish larvae used for the training and validation of the deep learning model for classification.)
- trainedModel.zip (Pretrained model.)

### Extended data

Zenodo: Qualitative image annotation plugins for Fiji - https://doi.org/10.5281/zenodo.4063891 ( Thomas, 2020).

This project contains the following extended data:

- Supplementary Figure 1: Detail of the input for the multi-class (dropdown) plugin.
- Supplementary Figure 2: Custom plugin for data-visualization as a pie chart in Fiji.
- Supplementary Figure 3: Visualizing data-distribution using sunburst charts in KNIME.
- Supplementary Figure 4: Training a deep learning model for image classification in KNIME using the generated annotations.
- Supplementary Figure 5: Detail of the Keras network learner KNIME node.
  Data are available under the terms of the Creative Commons Attribution 4.0 International license (CC-BY 4.0).

## Software availability

**Source codes, documentation and example workflows are available at:** https://github.com/LauLauThom/Fiji-QualiAnnotations.

**Archived source code at time of publication:** https://doi.org/10.5281/zenodo.4064118 ( Thomas, 2020).

**License:** GNU General Public License v3.

The plugins can be installed in Fiji by simply activating the *Qualitative Annotations* update site. Then the plugins are listed under the menu *Plugins > Qualitative Annotations*.

A pre-configured Fiji installation bundle for windows is also archived in the release section of the repository.

The following KNIME workflows and associated documentation README files are available in the subdirectory *KNIMEworkflows* under a Creative Commons Attribution 4.0 International License (CC-BY):

- View-Images-And-Annotations workflow

- Sunburst-chart-workflow (with csv of annotations for multi-cellular embryos)

- Deep-Learning – binary classifier – training workflow

- Deep-Learning – binary classifier – prediction workflow

- Deep-Learning – multi-class classifier – training workflow

- Deep-Learning – multi-class classifier – prediction workflow

## References

[ref-1] Bankhead P Loughrey MB Fernández JA: QuPath: Open source software for digital pathology image analysis. *Sci Rep.* 2017;7(1):16878. 10.1038/s41598-017-17204-5 29203879 PMC5715110

[ref-2] Berg S Kutra D Kroeger T: ilastik: interactive machine learning for (bio)image analysis. *Nat Methods.* 2019;16(12):1226–1232. 10.1038/s41592-019-0582-9 31570887

[ref-3] Berthold MR Cebron N Dill F: KNIME - the Konstanz information miner: version 2.0 and beyond. *ACM SIGKDD Explor Newsl.* 2009;11(1):26–31. 10.1145/1656274.1656280

[ref-4] de Chaumont F Dallongeville S Chenouard N: Icy: an open bioimage informatics platform for extended reproducible research.Focus on bioimage informatics. * Nat Methods.* 2012;9(7):690–696. 10.1038/nmeth.2075 22743774

[ref-6] Hollandi R Diósdi A Hollandi G: AnnotatorJ: an ImageJ plugin to ease hand annotation of cellular compartments.Ed. Lippincott-Schwartz, J. * Mol Biol Cell.* 2020;31(20):2179–2186. 10.1091/mbc.E20-02-0156 32697683 PMC7550707

[ref-8] Müller AC Guido S: Introduction to machine learning with Python: a guide for data scientists. First edition. ed. O’Reilly Media, Inc, Sebastopol, CA.2016. Reference Source

[ref-9] Pandey G Gehrig J Thomas L: Fluorescently-labelled zebrafish pronephroi + ground truth classes (normal/cystic) + trained CNN model (Version 1.0) [Data set]. *Zenodo.* 2020. 10.5281/zenodo.3997728

[ref-10] Pandey G Westhoff J Schaefer F: A Smart Imaging Workflow for Organ-Specific Screening in a Cystic Kidney Zebrafish Disease Model. *Int J Mol Sci.* 2019;20(6):1290. 10.3390/ijms20061290 30875791 PMC6471943

[ref-11] Schindelin J Arganda-Carreras I Frise E: Fiji: an open-source platform for biological-image analysis. *Nat Methods.* 2012;9(7):676–682. 10.1038/nmeth.2019 22743772 PMC3855844

[ref-12] Schneider CA Rasband WS Eliceiri KW: NIH Image to ImageJ: 25 years of image analysis. *Nat Methods.* 2012;9(7):671–675. 10.1038/nmeth.2089 22930834 PMC5554542

[ref-14] Thomas L: Qualitative image annotation plugins for Fiji (Version 1.0.2bis). *Zenodo.* 2020. 10.5281/zenodo.4064118

[ref-15] Thomas LS Gehrig J: ImageJ/Fiji ROI 1-click tools for rapid manual image annotations and measurements.2020. 10.17912/micropub.biology.000215 PMC7252406 32550514

[ref-17] Westhoff JH Steenbergen PJ Thomas LSV: *In vivo* High-Content Screening in Zebrafish for Developmental Nephrotoxicity of Approved Drugs. *Front Cell Dev Biol.* 2020;8:583. 10.3389/fcell.2020.00583 32754590 PMC7366291

