# Supplementary material for: Fiji plugins for qualitative image annotations: routine analysis and application to image classification
Source: F1000Res. 2021 Feb 12;9:1248. Originally published 2020 Oct 15. [Version 2] doi: 10.12688/f1000research.26872.2 (PMC8014705; doi:10.12688/f1000research.26872.2)
Supplement: Peer review history

# Peer review: Reviewer response for version 2

**DOI:** 10.5256/f1000research.54338.r81499

**Published:** 2021-03-31

**Authors:** Elisabet Teixido

**Affiliations:** 1 Toxicology Unit, University of Barcelona, Barcelona, Spain

The authors present a new set of Fiji plugins for the qualitative annotation of images in a single or multiple descriptive keywords. They also include the possibility to annotate outlined ROIs of the image and include some quantitative measurements using the defined menu in Fiji.

 There is an increasing need for future development of annotation tools in order to make the annotation process more efficient and they design a very useful tool for the annotation of 2D images. 

 The publication is well written and describes the plugin in detail, in addition there are some youtube tutorials that can help you understand and install the plugin in Fiji. I really appreciate the inclusion of some examples for the generated annotations, they include some analysis in ImageJ itself and also presented pipelines for data-visualization and a deep learning model for classification in KNIME. 

 I just have some comments and future suggestions for the tool. 

 Biomedical researchers are looking to use ontologies to support cross-laboratory data sharing and integration. It would be useful to be able to fetch categories from a file and not from a table of a set of images previously analysed. That way we may create common vocabulary annotation sets following ontology terms that can be shared across labs.

 The only current limitation of the tool is not been able to save coordinates of the ImageJ ROIs. It would be useful for shape analysis and also for building machine learning models to automatically identify for instance anatomical parts of an image.

Are the conclusions about the tool and its performance adequately supported by the findings presented in the article?

Yes

Is the rationale for developing the new software tool clearly explained?

Yes

Is the description of the software tool technically sound?

Yes

Are sufficient details of the code, methods and analysis (if applicable) provided to allow replication of the software development and its use by others?

Yes

Is sufficient information provided to allow interpretation of the expected output datasets and any results generated using the tool?

Yes

Reviewer Expertise:

Bioimage analysis, Toxicology

I confirm that I have read this submission and believe that I have an appropriate level of expertise to confirm that it is of an acceptable scientific standard.

# Peer review: Reviewer response for version 2

**DOI:** 10.5256/f1000research.54338.r79507

**Published:** 2021-02-26

**Authors:** Christian Tischer, Aliaksandr Halavatyi

**Affiliations:** 1 European Molecular Biology Laboratory, Heidelberg, Germany; 2 European Molecular Biology Laboratory (EMBL), Heidelberg, Germany

We now approve this article for indexing.

Are the conclusions about the tool and its performance adequately supported by the findings presented in the article?

Yes

Is the rationale for developing the new software tool clearly explained?

Yes

Is the description of the software tool technically sound?

Yes

Are sufficient details of the code, methods and analysis (if applicable) provided to allow replication of the software development and its use by others?

Yes

Is sufficient information provided to allow interpretation of the expected output datasets and any results generated using the tool?

Partly

Reviewer Expertise:

NA

We confirm that we have read this submission and believe that we have an appropriate level of expertise to confirm that it is of an acceptable scientific standard.

# Peer review: Reviewer response for version 1

**DOI:** 10.5256/f1000research.29676.r74544

**Published:** 2020-11-26

**Authors:** Christian Tischer, Aliaksandr Halavatyi

**Affiliations:** 1 European Molecular Biology Laboratory (EMBL), Heidelberg, Germany; 2 European Molecular Biology Laboratory, Heidelberg, Germany

The authors present an ImageJ plugin suite for the annotation of image planes and regions of interest (ROI) within image planes, using standard ImageJ technology such as results tables and the ROI manager. We think this is a very valuable addition to the ImageJ ecosystem as it is easy to use and very well integrated with current functionality that is already known to many users and developers.

 Below we have some specific comments which we hope could be helpful to further improve the publication and the plugins.

 We thank the authors for their efforts in implementing this great addition to the ImageJ ecosystem and hope that below suggestions are helpful!

 With kind regards,

 Christian Tischer and Aliaksandr Halavatyi

Suggestions for the publication

 We think the publication is nicely written and describes their plugins very well.

 The authors describe how the output of their plugins could be consumed by a machine learning workflow in KNIME. While the workflow in KNIME is very interesting and relevant we feel at the same time that it is slightly out of scope and we thus suggest moving this to the supplemental material. The reason is that we would expect the main readers of this publication and also the main users of the plugins to be familiar with the ImageJ ecosystem, but not necessarily with KNIME. In addition, if the final aim of the users is to execute a workflow in KNIME there may be other annotation tools available and the authors should clarify how their tool is superior to potential other solutions for image annotation.

 Along those lines, it would be great to also present (maybe also in the supplemental material) a follow up workflow that can be executed entirely within ImageJ. Specifically, it would be great to see an example of how the annotated ROIs could be used, e.g. to train a machine learning model. However, we don’t know how feasible this is and it is really just a suggestion.  

Technical issues and suggestions for the plugins

 There are hotkeys (F1, F2, …) available for the single class plugin, which is great as it speeds up annotation. However, we did not manage to have them working on MacOS (10.14.6). In addition, if one has many classes one would have to count the buttons to figure out which hotkey belongs to which class. We thus suggest that the hotkeys are either written on the category buttons or appears as pop-up messages when a user hovers over the corresponding button.

 Currently, one cannot add additional categories during the labelling process. We think it would be great to be able to (e.g., via a new button: [ Add category ] ), because images may contain phenotypes that one did not think about upfront.

 One can annotate the same image, slice and ROI multiple times, where each annotation will add another row to the table. As a consequence, the table contains conflicting annotations for the same image region. We think it would be (much) better if the annotation was replaced, at least optionally. 

 The possibility to continue an annotation by loading a table and renaming it to “Annotations” is a useful feature, but we think the current implementation has a limitation in a sense that the class names are not read from the table, but rather taken from the last annotation session. This can be an issue if users work on several annotations. We suggest adding an option to fetch the set of category names from the table.

 The logic of jumping to the next image slice in case of hyperstacks (4D or 5D data) is not completely obvious. We suggest adding a drop-down menu next to the “auto next slice” checkbox, where the user can select the dimension (z, c, or t) along which to automatically progress. 

 Currently, annotating a set of images that cannot be loaded into a hyperstack, e.g. because they may have different sizes or dimensionality, is possible but a bit tedious.  We suggest adding a modality where the user would select one folder on the disk and then the plugin would automatically open and close the images in the folder one-by-one, allowing them to be annotated one-by-one. The additional advantage of this modality also is that loading different images into a hyperstack is not something that every user may know how to achieve. We suggest adding [ previous ] and [ next ] buttons to this modality, allowing one to correct a previous annotation in case a mistake has been made.

 ImageJ ROIs are not a “cross platform standard”. In order to enable follow up workflows outside ImageJ it may be thus useful to store the bounding box coordinates for rectangular ROIs in the table.

 When clicking the Help button on one of the annotation UIs the UI was closing, which we think is probably a bug (tested on Mac).

Are the conclusions about the tool and its performance adequately supported by the findings presented in the article?

Yes

Is the rationale for developing the new software tool clearly explained?

Yes

Is the description of the software tool technically sound?

Yes

Are sufficient details of the code, methods and analysis (if applicable) provided to allow replication of the software development and its use by others?

Yes

Is sufficient information provided to allow interpretation of the expected output datasets and any results generated using the tool?

Partly

Reviewer Expertise:

Bioimage analysis

We confirm that we have read this submission and believe that we have an appropriate level of expertise to confirm that it is of an acceptable scientific standard, however we have significant reservations, as outlined above.

# Response

**Published:** 2021-01-29

**Authors:** Laurent Thomas

**Affiliations:** ACQUIFER Imaging GmbH, Germany

We thank the reviewers for their constructive criticism and helpful suggestions. Since the first manuscript version we implemented most of the previous suggestions to improve the plugins, which are readily available by updating the plugins in Fiji. We further address each point in details below.

  

- *The authors describe how the output of their plugins could be consumed by a machine learning workflow in KNIME. While the workflow in KNIME is very interesting and relevant we feel at the same time that it is slightly out of scope and we thus suggest moving this to the supplemental material. The reason is that we would expect the main readers of this publication and also the main users of the plugins to be familiar with the ImageJ ecosystem, but not necessarily with KNIME. In addition, if the final aim of the users is to execute a workflow in KNIME there may be other annotation tools available and the authors should clarify how their tool is superior to potential other solutions for image annotation.*

Following this observation, we have simplified the article such that the examples of visualization and analysis (including the deep learning workflows) are mentioned in the “Use cases” and summarized in the new Figure 4. Details about the deep learning implementation and individual workflow requirements is covered in Supplementary Figures (available on the Zenodo repository) and in the online documentation of the GitHub repository.

 Regarding annotation tools, there are several software packages available for this specific purpose of ground-truth annotations for supervised machine learning. However, as now explained in introduction they are sometimes not adapted to scientific image formats, while most of them are not integrated into user-oriented scientific image analysis software, and thus require additional installation or configuration. The qualitative annotations plugins instead can be easily installed in ImageJ/Fiji, which supports a large variety of scientific image formats, and is a familiar software environment for most life-scientists. Another advantage of the presented plugins is the support for multiple descriptive keywords per image instance, which is not systematically available with other software packages.

 Ground-truth category annotations for image-classification or object-detection is one possible application of the qualitative annotation tools. We chose KNIME to illustrate the training of an image-classifier, because it is a relatively accessible data-analysis platform for non-expert users, compared to a programming language. It supports advanced image-processing functionalities, was readily used for bioimage analysis and allows rapid prototyping and customization thanks to its interactive graphical user interface. However, more advanced users can surely reproduce this workflow in a programming language of their choice.

  

- *Along those lines, it would be great to also present (maybe also in the supplemental material) a follow up workflow that can be executed entirely within ImageJ. Specifically, it would be great to see an example of how the annotated ROIs could be used, e.g. to train a machine learning model. However, we don’t know how feasible this is and it is really just a suggestion. *

It is potentially possible to train a machine learning or deep learning model directly in ImageJ/Fiji e.g. thanks to respectively the Weka or TensorFlow integration in Fiji. However, this would represent a significative amount of work, and would not offer as much flexibility as the reported KNIME workflows for deep learning, which can be rapidly adapted in KNIME’s graphical user-interface.

 As an illustration of use case in Fiji, we propose instead an additional plugin for data-visualization relying on the JFreeChart library (available with Fiji). This new plugin generates a pie chart from a table column, to visualize the category distribution. It is available in the same menu “Qualitative Annotation” under “PieChart from category column”– (see Figure 5.B and supplementary figure 2).

 The plugins support any column of data from a table opened in Fiji and is macro recordable. It is thus not limited to annotation tables generated with the presented plugins. To our knowledge, the pie chart visualization was not previously available to users via the Fiji menus or plugins. Therefore, we believe that it can be an interesting complement to the annotation plugins.  

***Technical issues and suggestions for the plugins***

- *There are hotkeys (F1, F2, …) available for the single class plugin, which is great as it speeds up annotation. However, we did not manage to have them working on MacOS (10.14.6)*

At the time of the fist manuscript version, the plugins were only tested on windows but are expected to run similarly across platforms. Hotkeys might be an exception, for instance on Linux we also observe a different behaviour (see Issue #9 · LauLauThom/Fiji-QualiAnnotations (github.com)).

 We don’t have a MacOS system at hand for testing, but we invite the reviewers to follow up in the dedicated issue thread on GitHub mentioned above.

 The hotkeys are sometimes not responsive also on Windows. Clicking the plugin window or one of the buttons usually allows reactivating the hotkey functionality.

 That said, we believe this is not a major issue preventing the use of the plugins, and we hope to fix it in later versions of the plugins.

   

- *[..] if one has many classes one would have to count the buttons to figure out which hotkey belongs to which class. We thus suggest that the hotkeys are either written on the category buttons or appears as pop-up messages when a user hovers over the corresponding button.*

We have updated the button plugin with buttons from the java swing package (previously java awt buttons), which support pop-up messages. The associated hotkey is thus now displayed when the mouse is hovered over a category button. With this new button class, the layout of the GUI might be slightly impaired when a new button is added by clicking the “Add new category”. When this happens, the window can be resized manually to make sure all GUI elements fits in the window. We will fix this issue in later plugin versions if we can identify the source of the problem.

  

- *Currently, one cannot add additional categories during the labelling process. We think it would be great to be able to (e.g., via a new button: [ Add category ] ), because images may contain phenotypes that one did not think about upfront.*

We added a “Add new category” button to the *single class (button)* and *multi-class (checkboxes)* plugins, which allows updating the plugin interface with new categories on the fly.

 We did not add this functionality to the dropdown plugin, which would require providing multiple items (label and list of choices). However, with any of the annotation plugins, the plugin interface can be closed and restarted while keeping the current annotation table opened in ImageJ/Fiji. Although not as straightforward as the “Add new category” option, the annotation can thus be interrupted to update the graphical interface and resumed at a later time without losing information.

  

- *One can annotate the same image, slice and ROI multiple times, where each annotation will add another row to the table. As a consequence, the table contains conflicting annotations for the same image region. We think it would be (much) better if the annotation was replaced, at least optionally. *

We have considered the possibility to add an option (checkbox) to replace previous annotations of identical image or ROI (see dedicated branch on GitHub). However, when “run Measure” is selected, a table row is anyway added to the result table with the quantitative measurements, before the descriptive keywords are added to the table row. Accounting for this alternative behaviour would further complicate the code (see function defaultActionSequence() ). A more elegant solution would be to have a way to store the measurement into a variable first, which is apparently not the case with the current ImageJ1 package.

 We thus might address this issue in later releases of the tools.

  

- *The possibility to continue an annotation by loading a table and renaming it to “Annotations” is a useful feature, but we think the current implementation has a limitation in a sense that the class names are not read from the table, but rather taken from the last annotation session. This can be an issue if users work on several annotations. We suggest adding an option to fetch the set of category names from the table.*

We added an option to fetch the categories names from a table currently opened in ImageJ/Fiji.

 If the table contains a column name “Category” then the set of categories is initialized from this column. Otherwise, the set of categories is initialized from the column headers, by excluding the headers for the measurement and file information columns. However, the number of categories will still be taken into account.

 Besides, with ImageJ versions above 1.53g, the annotations will be appended to/read from any active table window. Below this version, the annotations are appended to/read from table windows entitled “Annotations” or “Annotations.csv”.

  

- *The logic of jumping to the next image slice in case of hyperstacks (4D or 5D data) is not completely obvious. We suggest adding a drop-down menu next to the “auto next slice” checkbox, where the user can select the dimension (z, c, or t) along which to automatically progress. *

As suggested, we added a dropdown menu next to the “Auto next slice” checkbox to specify the dimension to explore with hyperstacks. This dropdown menu is shown when “stack” is selected as browsing mode in the initial configuration window (see below).

  

- *Currently, annotating a set of images that cannot be loaded into a hyperstack, e.g. because they may have different sizes or dimensionality, is possible but a bit tedious.  We suggest adding a modality where the user would select one folder on the disk and then the plugin would automatically open and close the images in the folder one-by-one, allowing them to be annotated one-by-one. The additional advantage of this modality also is that loading different images into a hyperstack is not something that every user may know how to achieve. We suggest adding [ previous ] and [ next ] buttons to this modality, allowing one to correct a previous annotation in case a mistake has been made.*

We added a new setting “Browsing mode” in the initial configuration window, which can be set to “stack” or “directory”. The “stack” mode corresponds to the previously available modality and is well adapted to the annotations of image slices in stacks or hyperstacks.

 With the “directory” mode, ticking the “Auto next slice/image file” will switch to the next image file in the directory of the currently opened image file. This allows annotating a list of files in a directory as proposed. In “directory” browsing mode the annotation interface also has “previous” and “next image file” buttons, as suggested.

  

- *ImageJ ROIs are not a “cross platform standard”. In order to enable follow up workflows outside ImageJ it may be thus useful to store the bounding box coordinates for rectangular ROIs in the table.*

Bounding box coordinates for ROIs can be readily recovered by activating the “Bounding Rectangle” option in Analyze > Set measurements and activating the “run Measure” option in the plugin configuration.

 For other ROI shapes, such as polygon and free shapes, the number of summits/coordinates is variable. There is thus the option to have on column per coordinates, which is not ideal as it can potentially yield lots of columns. The second option is to have a single column containing the coordinates as a list or another type of collection. However, there is no real standard convention for polygons and other free ROI shapes coordinates encoding. Some further discussion would thus be needed before implementing such functionality (in a dedicated GitHub issue for instance).

  

- *When clicking the Help button on one of the annotation UIs the UI was closing, which we think is probably a bug (tested on Mac).*

It is expected that clicking the help button should close the UI, but it should also open the page of the GitHub repository in the default browser. If this is not the case this might be an OS-dependant error, like for the hotkeys. We have opened a dedicated issue thread on GitHub ( Issue #22 · LauLauThom/Fiji-QualiAnnotations (github.com).

# Peer review: Reviewer response for version 1

**DOI:** 10.5256/f1000research.29676.r73675

**Published:** 2020-11-13

**Authors:** Jan Eglinger

**Affiliations:** 1 Friedrich Miescher Institute for Biomedical Research (FMI), Basel, Switzerland

The authors introduce a new set of Fiji plugins that allows user-friendly annotation of images in two ways: 1) tagging entire images with one or several keywords/classes, and 2) tagging selected regions within an image.

 Thanks to the integration with the Fiji/ImageJ framework (installation via an ImageJ update site, graphical user interface using the built-in functionality provided by Fiji), the plugins are indeed simple to install and use, and simplify the process to get tabular data from a manual annotation task.

 In the introduction, the authors state that there is "no widespread solution available [...] for the assignment of multiple descriptive keywords to images". In my opinion, this statement demonstrates a lack of research, as a simple online search for "image annotation tool" reveals many solutions used in the field of Computer Vision to annotate ground truth for machine learning datasets (both for image classification and for bounding box annotations). While some of these tools might be more commonly used than others, I believe they should have at least included a few of them in a thorough comparison, such as e.g. LabelMe [1], MakeSense.AI [2], LabelImg [3], VGG Image Annotator [4], or Scalabel [5]. The only other available tool they cite is ACQUIFER Manual Annotation Tool, a software created by (partially) the same authors, available on request only.

 Nevertheless, the set of plugins introduced here certainly fills a gap for users of Fiji/ImageJ in particular, which means a lot of scientists using it to answer biological questions with (microscopy) image data, who will welcome a way to generate annotation data from within a framework that they already use and know.

 Specifically for biological applications however, I see a potential limitation of the use of this plugin. With modern microscopy datasets often being multi-dimensional, region annotations (bounding-box or pixel-wise) would benefit from being 3-dimensional to reflect a volume of interest. By being tied very much to the legacy user interface of Fiji (built-in ROI support is 2D only), the Qualitative Annotations plugin will be of very limited use for true 3D applications, since any annotation can only be done slice-wise, in the current state. Other plugins, such as the 3D ImageJ Suite, offer support for 3-dimensional object annotations, but lack an easy way to add keywords/class annotations.

 Another current limitation of the Qualitative Annotations plugin is the absence of ways to review and/or refine an annotation after it has been added to the results table.

 Lastly, since the authors include KNIME workflows in their article to illustrate further processing of the annotation data (e.g. for training of machine learning models), I am surprised that they didn't also explore ways to do the manual annotation within a KNIME workflow, in order to include the annotation process in their "complete pipeline for image classification".  

 The KNIME Image Processing [6] extension offers the 'Interactive Annotator' and 'Interactive Labeling Editor' nodes that can be used to annotate image regions. For interactive sequential classification of images, the 'Active Learning Loop' nodes offer an excellent alternative. These options should be mentioned for comparison when discussing the performance of the Qualitative Annotations tools introduced here.

Are the conclusions about the tool and its performance adequately supported by the findings presented in the article?

Partly

Is the rationale for developing the new software tool clearly explained?

Partly

Is the description of the software tool technically sound?

Yes

Are sufficient details of the code, methods and analysis (if applicable) provided to allow replication of the software development and its use by others?

Yes

Is sufficient information provided to allow interpretation of the expected output datasets and any results generated using the tool?

Yes

Reviewer Expertise:

Image Processing, Software Development, Microscopy

I confirm that I have read this submission and believe that I have an appropriate level of expertise to confirm that it is of an acceptable scientific standard, however I have significant reservations, as outlined above.

# Response

**Published:** 2021-01-29

**Authors:** Laurent Thomas

**Affiliations:** ACQUIFER Imaging GmbH, Germany

Dear Jan, thank you for your feedback. The tools are indeed mostly targeting the annotation of 2D images, or from image slices in multi-dimensional images. They are not designed to address qualitative annotations of 3D volumes, which as you pointed out have limited support in ImageJ/Fiji.

*In the introduction, the authors state that there is "no widespread solution available [...] for the assignment of multiple descriptive keywords to images". In my opinion, this statement demonstrates a lack of research, as a simple online search for "image annotation tool" reveals many solutions used in the field of Computer Vision to annotate ground truth for machine learning datasets (both for image classification and for bounding box annotations). While some of these tools might be more commonly used than others, I believe they should have at least included a few of them in a thorough comparison, such as e.g. LabelMe [1], MakeSense.AI [2], LabelImg [3], VGG Image Annotator [4], or Scalabel [5].*

 Regarding other image annotations solutions mentioned above, most of them target RGB colour images and thus are not well adapted to scientific images or file format (e.g 16-bit multi-dimensional tiff). Besides, they mostly target single-category annotations of images or images regions for classification, object-detection or semantic segmentation and usually do not allow the assignment of multiple qualitative keywords to a single image instance. Moreover, these are often standalone software, and thus for non-experts likely not as accessible as integrated solutions in bioimage analysis software such as ImageJ/Fiji. We have reformulated the introduction to include a brief comparison with existing solutions and their limitations. Although the plugins presented in this article can indeed be used for the tasks mentioned above, we still believe that the annotations with multiple keywords was a truly missing functionality and can be of major interest to describe complex biological phenotypes.

*Another current limitation of the Qualitative Annotations plugin is the absence of ways to review and/or refine an annotation after it has been added to the results table.*

 Regarding this limitation, again due to its tight integration in ImageJ/Fiji, individual table cells of the result table cannot be edited interactively. However, if a mistake was done while annotating, the concerned table rows can be selected and deleted, and the annotation repeated for the images. We have added a sentence in the paragraph implementation mentioning this possibility. The table could also be edited using the macro language, although it is likely not a probable use-case.

 After annotations, the csv file containing the results can be edited in any software supporting csv such as spreadsheet software or text editors.

*Lastly, since the authors include KNIME workflows in their article to illustrate further processing of the annotation data (e.g. for training of machine learning models), I am surprised that they didn't also explore ways to do the manual annotation within a KNIME workflow, in order to include the annotation process in their "complete pipeline for image classification".*

*The KNIME Image Processing [6] extension offers the 'Interactive Annotator' and 'Interactive Labeling Editor' nodes that can be used to annotate image regions. For interactive sequential classification of images, the 'Active Learning Loop' nodes offer an excellent alternative. These options should be mentioned for comparison when discussing the performance of the Qualitative Annotations tools introduced here.*

 KNIME indeed offers powerful functionalities for ground-truth annotations of image regions or image category, however the annotations are here also limited to a single category per ROI or image. Besides, despite its advanced data and image-analysis functionalities, we believe that it is not the most intuitive software for interactive data-annotation, especially for users not already familiar with KNIME. Still, we have included KNIME in the list of existing annotation solutions compatible with bioimages, in the introduction.
